# Supplementary material for: K-PAM: a unified platform to distinguish Klebsiella species K- and O-antigen types, model antigen structures and identify hypervirulent strains
Source: Sci Rep. 2020 Oct 7;10:16732. doi: 10.1038/s41598-020-73360-1 (PMC7541508; doi:10.1038/s41598-020-73360-1)
Supplement: Supplementary file 1 — Supplementary Information 1. [file 41598_2020_73360_MOESM1_ESM.docx]

**K-PAM: A unified platform to distinguish *Klebsiella* species K- and O-antigen types, model antigen structures and identify hypervirulent strains**

L Ponoop Prasad Patro^†^, Karpagam Uma Sudhakar^†^ and Thenmalarchelvi Rathinavelan*

**Supplementary Figures for K-PAM**

| **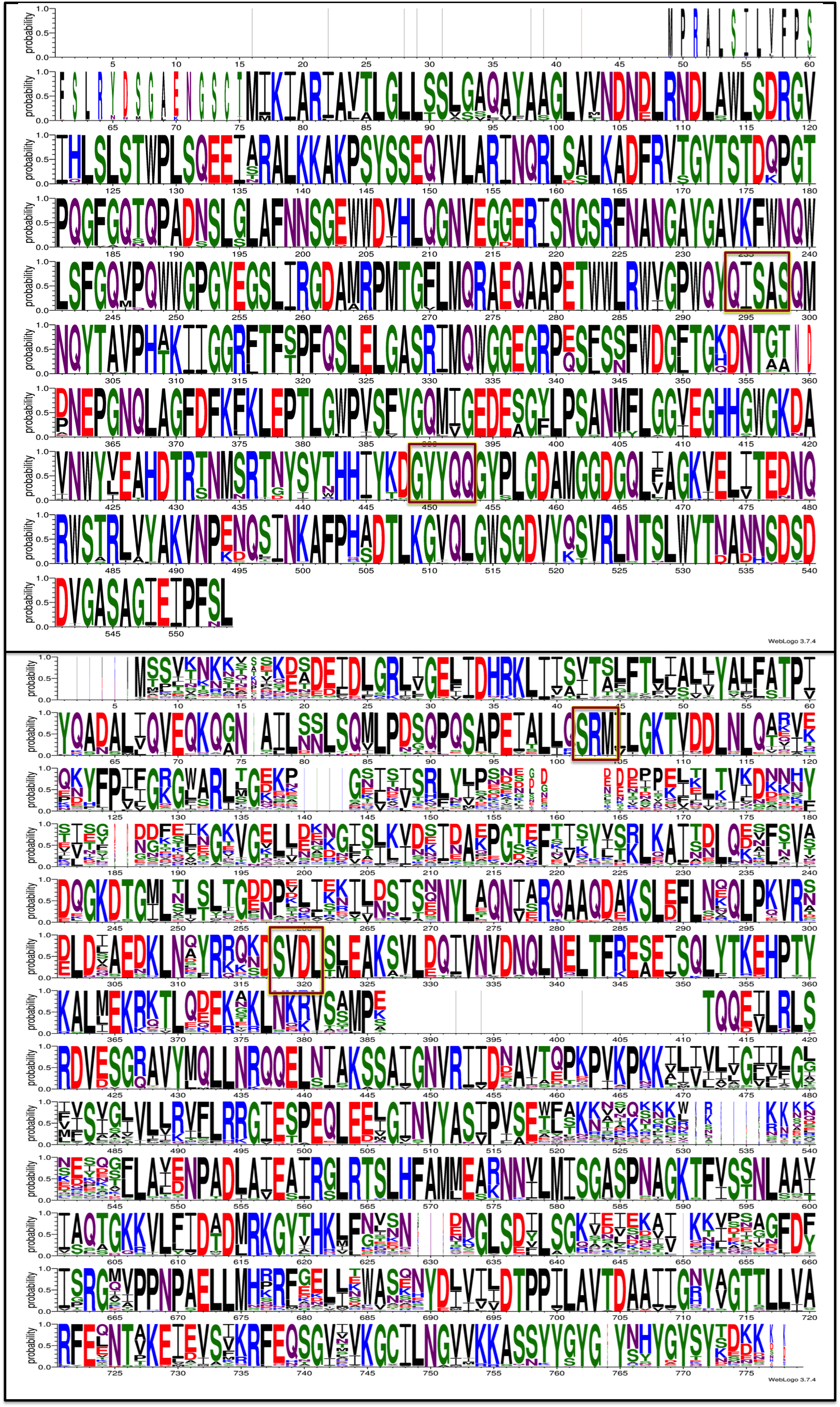** |
| --- |
| **Figure S1.** The amino acids sequence logo built using the multiple sequence alignment of 157 Wzi (**Top**) and 163 Wzc (**Bottom**) non-redundant protein sequences that are used in the creation of local database. “QISAS” & “GYYQQ” marker motifs used in the fragmentation of Wzi sequences (N-terminal, middle and C-terminal regions) and “SRM” & “SVDL” marker motifs used in the fragmentation of Wzc sequences are boxed. Note that the region falls between “SRM” & “SVDL” in Wzc corresponds to the periplasmic region of the protein that interacts with Wza to transport the K-antigens. |

| **(i)**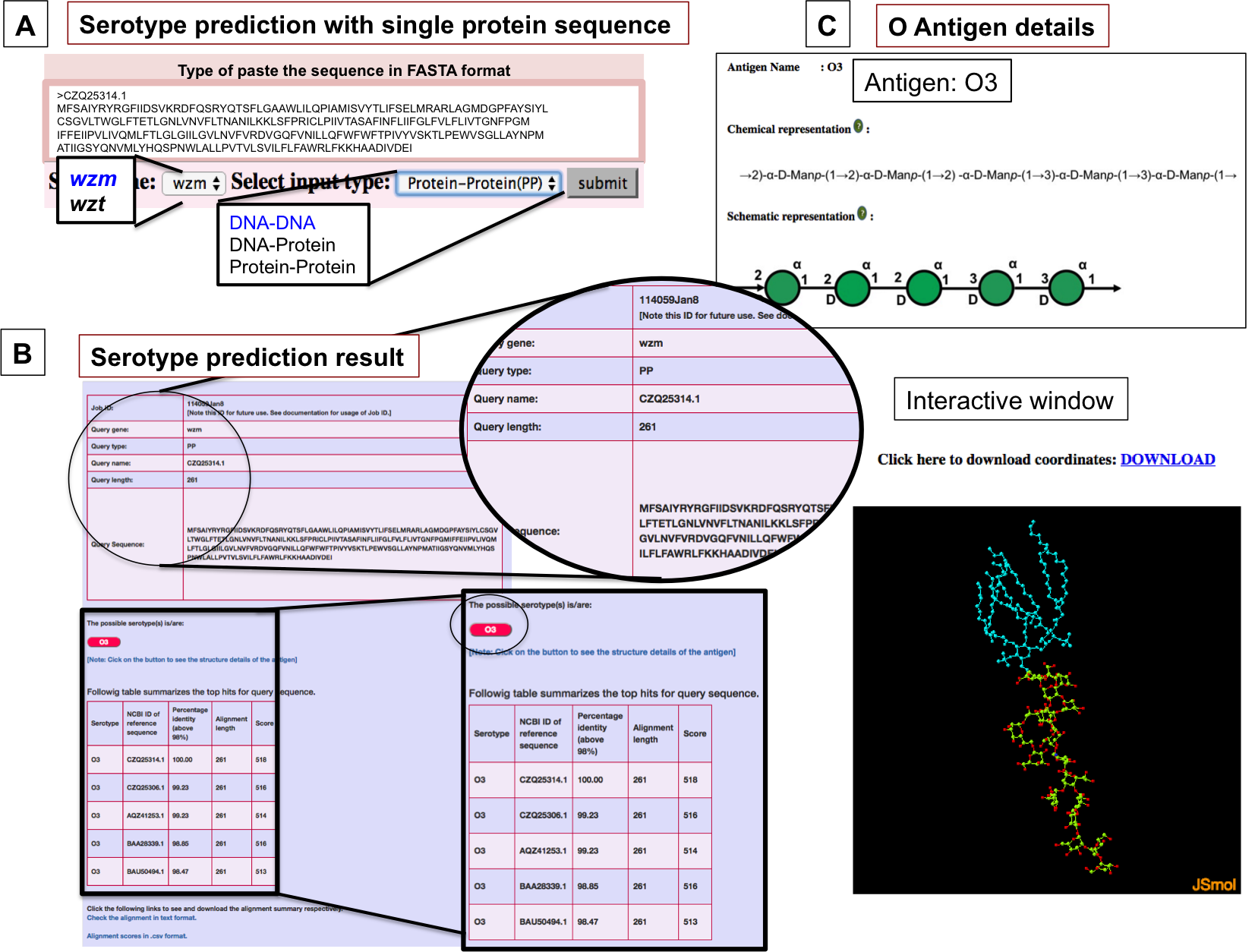 |
| --- |
| **(ii)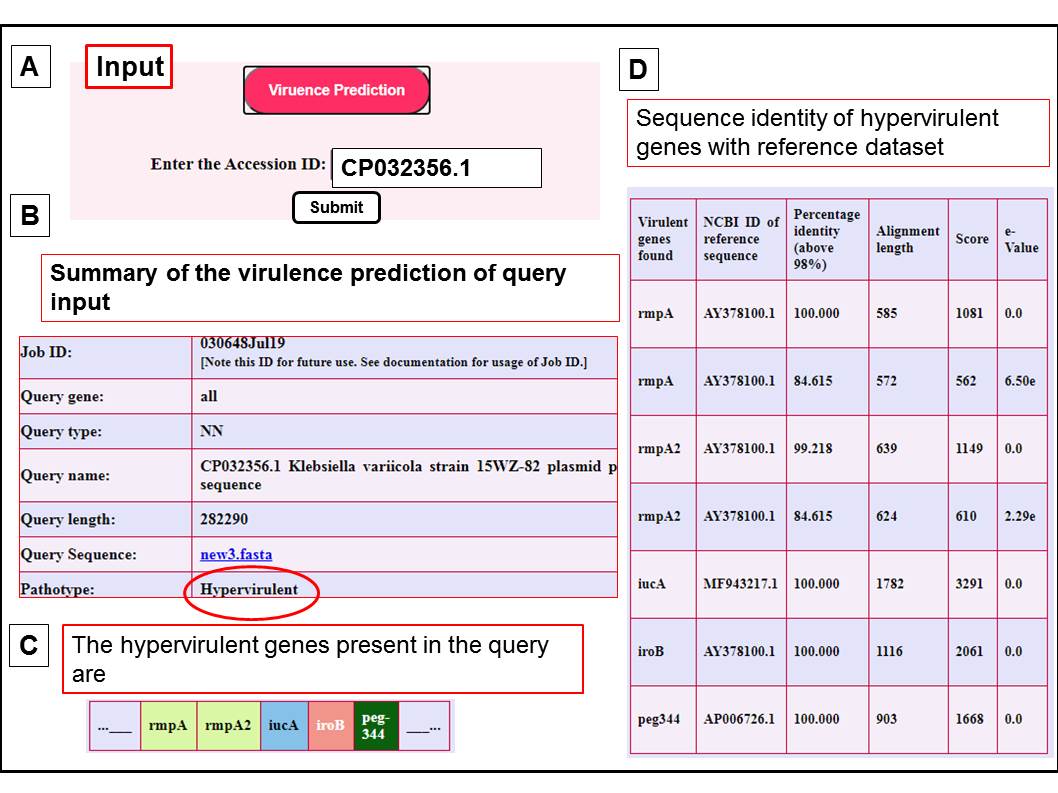** |
| **Figure S2.** The graphical user interface of K-PAM explaining (i) the O-type prediction and (ii) the hypervirulent strain identification. **(i)** The example shown here illustrates the *Klebsiella* O-type prediction using the NCBI accession number CZQ25314.1 that correspond to Wzm. A) The input box, in which, Wzm query sequence is submitted. The options, “Wzm” and “Protein-Protein” are selected. B) O-type prediction by K-PAM is summarized in the result page that contains the reference job ID, input sequence information and the predicted O-type. The clickable button (magenta button, circled) will lead to the detailed information about the antigen structure. C) The schematic and chemical representations of the predicted antigen along with the JSmol interactive view. **(ii)** An example illustrating the hypervirlent strain identification process followed in K-PAM by considering the hypervirulent *Klebsiella* *variicola* clinical strain as a test case (Genbank ID CP032356.1). |

| 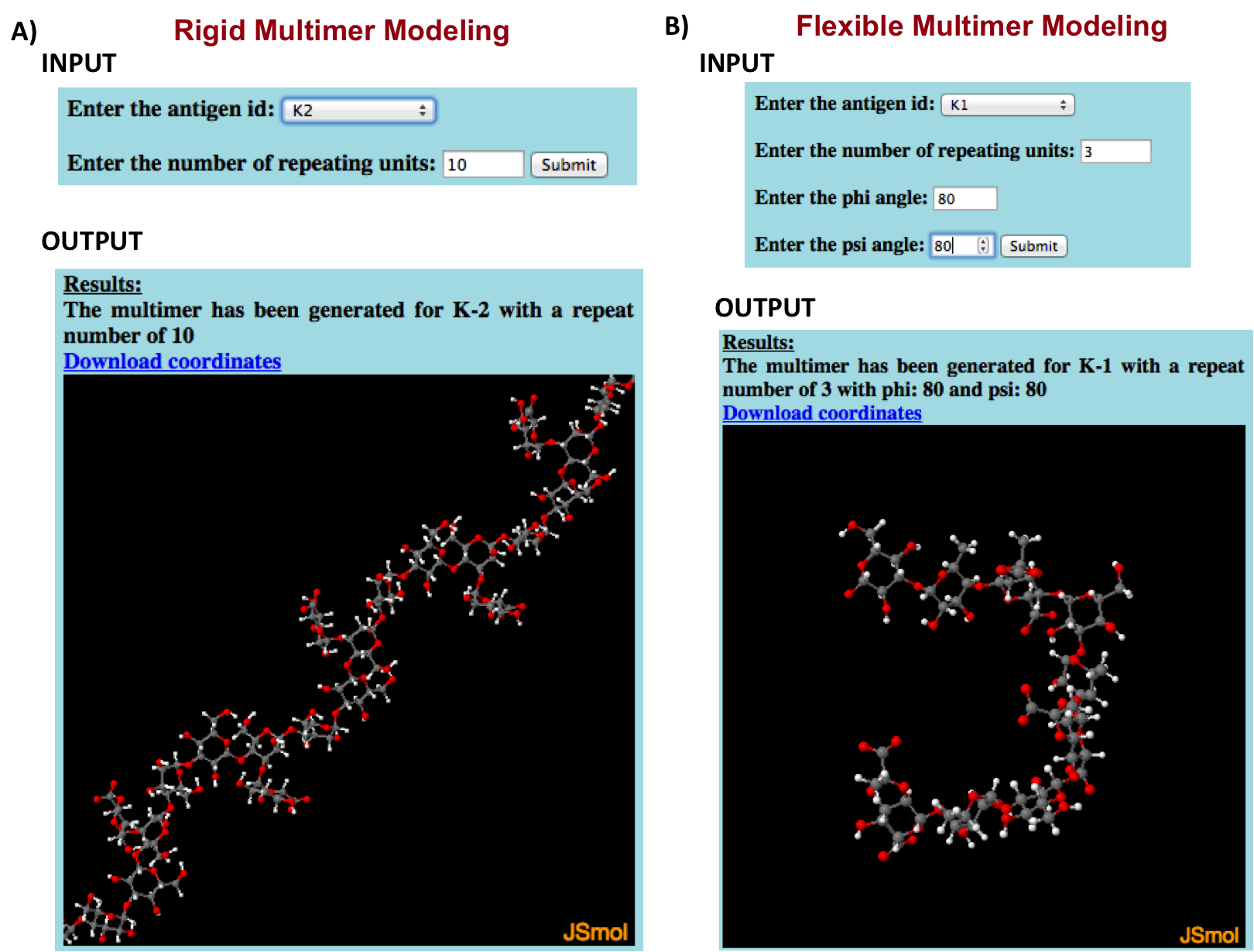 |
| --- |
| **Figure S3.** K-antigen multimer generation options. (A) Rigid multimer modeling (RMM) and (B) flexible multimer modeling (FMM) that help the user to generate the K-antigen multimers. |
