# Supplementary material for: K-PAM: a unified platform to distinguish Klebsiella species K- and O-antigen types, model antigen structures and identify hypervirulent strains
Source: Sci Rep. 2020 Oct 7;10:16732. doi: 10.1038/s41598-020-73360-1 (PMC7541508; doi:10.1038/s41598-020-73360-1)
Supplement: Supplementary file 2 — Supplementary Information 2. [file 41598_2020_73360_MOESM2_ESM.docx]

**K-PAM: A unified platform to distinguish *Klebsiella* species K- and O-antigen types, model antigen structures and identify hypervirulent strains**

L Ponoop Prasad Patro^†^, Karpagam Uma Sudhakar^†^ and Thenmalarchelvi Rathinavelan*

**Table S1.** The serotype prediction reliability calculated for (A) CPS locus proteins (for K-type) and, (B) Wzm and Wzt proteins (for O-type) by using the **Equation 1** (see the “Methodology” section in the main text). The average reliability score calculated by using the **Equation 2** (see the “Methodology” section in the main text) is given in the bottom row. **(A)** The reliability scores (column 3^rd^ to 11^th^) are color-coded as represented in the scale below. “NA” in cells stands for not applicable as, WbaP and WcaJ are mutually exclusive (either one of them is present). “NF” is mentioned for the cases where the particular protein/gene sequences for the corresponding K-type are not available. The column with title “Wzi_fbp” correspond to the reliability scores obtained after applying the fragment based approach for Wzi sequences (see the Results and Discussion section in the main text). **(B)** The reliability score (column 4^th^ to 5^th^) are color-coded as represented in the scale below.

**(A)**


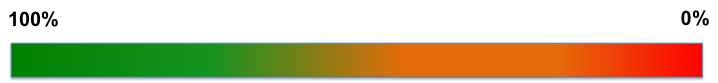


| **K-type** | **Genbank ID or Reference ID** | **Reliability score in %** | | | | | | | | |
| --- | --- | --- | --- | --- | --- | --- | --- | --- | --- | --- |
|  |  | **Wzi** | **Wzi_fbp** | **Wza** | **Wzb** | **Wzc** | **WbaP** | **Wzx** | **Wzy** | **WcaJ** |
| K1 | LT174541 | 50 | 100 | 100 | 50 | 100 | NA | 100 | 100 | 50 |
| K2 | AB371296 | 25 | 100 | 25 | 100 | 100 | NA | 100 | 100 | 100 |
| K3 | LT174553 | 5.55 | 100 | 100 | 100 | 100 | 100 | 100 | 100 | NA |
| K4 | AB924548 | 50 | 50 | 33.33 | 50 | 100 | NA | 100 | 100 | 50 |
| K5 | AB289645 | 100 | 100 | 100 | 100 | 100 | NA | 100 | 100 | 100 |
| K6 | AB924549 | 50 | 100 | 100 | 100 | 100 | NA | 100 | 100 | 100 |
| K7 | AB924550 | 5.88 | 50 | 100 | 100 | 100 | NA | 100 | 100 | 100 |
| K8 | AB924551 | 14.28 | 50 | 100 | 100 | 100 | NA | 100 | 100 | 100 |
| K9 | AB371293 | 2.94 | 100 | 50 | 50 | 50 | 100 | 100 | 100 | NA |
| K10 | AB924552 | 5.55 | 100 | 100 | 100 | 100 | 100 | 100 | 100 | NA |
| K11 | LT174533 | 50 | 100 | 50 | 100 | 100 | NA | 100 | 100 | 100 |
| K12 | AB924554 | 3.22 | 33.33 | 100 | 100 | 100 | 100 | 100 | 100 | NA |
| K13 | AB924555 | 100 | 100 | 33.33 | 100 | 100 | NA | 100 | 100 | 100 |
| K14 | AB371294 | 50 | 100 | 33.33 | 100 | 100 | NA | 100 | 100 | 100 |
| K15 | LT174536 | 4.34 | 20 | 25 | 50 | 100 | 100 | 100 | 100 | NA |
| K16 | AB742228 | 50 | 100 | 100 | 100 | 100 | NA | 100 | 100 | 100 |
| K17 | AB924557 | 20 | 100 | 100 | 100 | 100 | NA | 100 | 100 | 100 |
| K18 | AB924558 | 3.44 | 33.33 | 100 | 100 | 100 | 100 | 100 | 100 | NA |
| K19 | AB924559 | 5.26 | 33.33 | 100 | 100 | 100 | 100 | 100 | 100 | NA |
| K20 | AB289648 | 5.88 | 100 | 100 | 100 | 100 | 100 | 100 | 100 | NA |
| K21 | AB924560 | 4.76 | 100 | 50 | 50 | 50 | 100 | 50 | 50 | NA |
| K22 | AB819893 | 16.66 | 50 | 25 | 50 | 50 | NA | 50 | 50 | 50 |
| K23 | AB742229 | 50 | 50 | 25 | 100 | 100 | NA | 100 | 100 | 100 |
| K24 | AB924562 | 3.12 | 50 | 25 | 100 | 100 | NA | 100 | 100 | 100 |
| K25 | AB924563 | 14.28 | 100 | 100 | 100 | 100 | NA | 100 | 100 | 100 |
| K26 | AB924564 | 33.33 | 50 | 100 | 100 | 100 | 100 | 100 | 100 | NA |
| K27 | AB924565 | 3.84 | 33.33 | 100 | 100 | 100 | 100 | 100 | 100 | NA |
| K28 | AB924566 | 14.28 | 100 | 100 | 100 | 100 | NA | 100 | 100 | 100 |
| K29 | AB924567 | 33.33 | 50 | 100 | 100 | 100 | 100 | 100 | NA | NA |
| K30 | AB924568 | 16.66 | 50 | 50 | 50 | 50 | NA | 100 | 100 | 100 |
| K31 | AB924569 | 16.66 | 100 | 100 | 100 | 100 | NA | 100 | 100 | 100 |
| K32 | AB924570 | 100 | 100 | 100 | 100 | 100 | 100 | 100 | 100 | NA |
| K33 | AB924571 | NA | NA | 100 | 100 | 100 | NA | 100 | 100 | 100 |
| K34 | AB924572 | 100 | 100 | 100 | 100 | 100 | NA | NA | 100 | 100 |
| K35 | AB924573 | 100 | 100 | 100 | 100 | 100 | NA | 100 | 100 | 100 |
| K36 | AB924574 | 100 | 100 | 100 | 100 | 100 | 100 | 100 | 100 | NA |
| K37 | AB819894 | 14.28 | 50 | 25 | 50 | 50 | NA | 50 | 50 | 50 |
| K38 | AB924576 | 5.55 | 100 | 50 | 100 | 100 | 100 | 100 | 100 | NA |
| K39 | LT174552 | 5.26 | 100 | 20 | 100 | 100 | NA | 100 | 100 | 100 |
| K40 | AB924577 | NA | NA | 100 | 50 | 100 | 50 | 100 | 100 | NA |
| K41 | AB924578 | 100 | 100 | 100 | 100 | 100 | 100 | 100 | 100 | NA |
| K42 | AB924579 | 100 | 100 | 100 | 100 | 100 | 100 | 100 | 100 | NA |
| K43 | AB924580 | 5 | 50 | 100 | 100 | 100 | 100 | 100 | 100 | NA |
| K44 | AB924581 | 100 | 100 | 100 | 100 | 100 | NA | 100 | 100 | 100 |
| K45 | AB924582 | 100 | 100 | 25 | 50 | 50 | NA | 100 | 100 | 100 |
| K46 | AB924583 | 5 | 100 | 33.33 | 100 | 100 | 100 | 100 | 100 | NA |
| K47 | AB924584 | 3.57 | 50 | 33.33 | 100 | 100 | 100 | 100 | 100 | NA |
| K48 | AB924585 | 100 | 100 | 100 | 100 | 100 | NA | 100 | 100 | 100 |
| K49 | AB924586 | 2.7 | 100 | 100 | 100 | 100 | 100 | 100 | 100 | NA |
| K50 | AB924587 | 3.44 | 50 | 100 | NA | NA | 100 | NA | NA | NA |
| K51 | AB924588 | 3.84 | 20 | 25 | 100 | 100 | 100 | 100 | 100 | NA |
| K52 | AB924589 | 3.84 | 20 | 25 | 50 | 100 | 100 | 100 | 100 | NA |
| K53 | AB924590 | 4.76 | 100 | 100 | 100 | 100 | 100 | 100 | 100 | NA |
| K54 | AB924591 | 100 | 100 | 100 | 100 | 100 | NA | 100 | 100 | 33.33 |
| K55 | AB924592 | 4 | 100 | 100 | 100 | 100 | NA | 100 | 100 | 100 |
| K56 | AB924593 | 33.33 | 100 | 50 | 100 | 100 | 100 | 100 | 100 | NA |
| K57 | AB334776 | 3.84 | 33.33 | 33.33 | 100 | 100 | 100 | 100 | 100 | NA |
| K58 | AB924595 | 50 | 100 | 100 | 100 | 100 | NA | 100 | 100 | 50 |
| K59 | AB924596 | 100 | 100 | 100 | 100 | 100 | NA | 100 | 100 | 100 |
| K60 | AB924597 | 3.22 | 50 | 100 | 100 | 100 | NA | 100 | 100 | 100 |
| K61 | AB924598 | 3.7 | 33.33 | 100 | 100 | 100 | NA | 100 | 100 | 100 |
| K62 | AB371295 | 4 | 50 | 100 | 100 | 100 | 100 | 100 | 100 | NA |
| K63 | AB924599 | 3.22 | 25 | 33.33 | 100 | 100 | 100 | 100 | 100 | NA |
| K64 | AB924600 | 50 | 100 | 100 | 100 | 100 | NA | 100 | 100 | 100 |
| K65 | AB924601 | 50 | 50 | 100 | 100 | 100 | NA | 100 | 100 | 100 |
| K66 | AB924602 | 33.33 | 50 | 100 | 100 | 100 | 100 | 100 | 100 | NA |
| K67 | AB924603 | 100 | 100 | 100 | 100 | 100 | NA | 100 | 100 | 100 |
| K68 | AB924604 | 100 | 100 | 100 | 100 | 100 | 100 | 100 | 100 | NA |
| K69 | AB924605 | 100 | 100 | 100 | 100 | 100 | NA | 100 | 100 | 100 |
| K70 | AB924606 | 25 | 100 | 100 | 100 | 100 | 100 | 100 | 100 | NA |
| K71 | AB924607 | 9.09 | 100 | 25 | 100 | 100 | NA | 100 | NA | 100 |
| K72 | AB924608 | 100 | 100 | 100 | 100 | 100 | NA | 100 | 100 | 100 |
| K74 | AB924609 | 25 | 33.33 | 100 | 100 | 100 | 100 | 100 | 100 | NA |
| K79 | AB924610 | 100 | 100 | 100 | 100 | 100 | 100 | 100 | 100 | NA |
| K80 | AB924611 | 11.11 | 100 | 100 | 100 | 100 | 100 | 100 | 100 | NA |
| K81 | AB924612 | 4 | 33.33 | 16.66 | 100 | 100 | 100 | 100 | 100 | NA |
| K82 | AB924613 | 100 | 100 | 100 | 100 | 100 | NA | 100 | 100 | 100 |
| KL103 | LT174574 | 4.16 | 100 | 100 | 100 | 100 | 100 | 100 | 100 | NA |
| KL105 | LT174575 | 2.56 | 100 | 100 | 100 | 100 | 100 | 100 | 100 | NA |
| KL106 | LT174576 | 100 | 100 | 33.33 | 100 | 100 | NA | 100 | 100 | 100 |
| KL108 | LT174577 | 100 | 100 | 100 | 100 | 100 | NA | 100 | NA | 100 |
| KL109 | LT174578 | 100 | 100 | 100 | 100 | 100 | 100 | 100 | NA | NA |
| KL110 | LT174579 | 4 | 33.33 | 50 | 100 | 100 | 100 | 100 | NA | NA |
| KL111 | LT174580 | 16.66 | 33.33 | 100 | 100 | 100 | NA | 100 | 100 | 100 |
| KL112 | LT174581 | 7.14 | 100 | 100 | 100 | 100 | 100 | 100 | 100 | NA |
| KL113 | LT174582 | 50 | 100 | 100 | 100 | 100 | NA | 100 | NA | 33.33 |
| KL114 | LT174583 | 20 | 100 | 100 | 100 | 100 | 100 | 100 | 100 | NA |
| KL115 | LT174584 | 4.76 | 50 | 100 | 100 | 100 | 100 | 100 | 100 | NA |
| KL116 | LT174585 | 9.09 | 100 | 50 | 100 | 100 | 100 | 100 | NA | NA |
| KL117 | LT174586 | 100 | 100 | 100 | 100 | 100 | 100 | 100 | 100 | NA |
| KL118 | LT174587 | 33.33 | 100 | 100 | 100 | 100 | 100 | 100 | NA | NA |
| KL119 | LT174588 | 100 | 100 | 100 | 100 | 100 | NA | 100 | 100 | 100 |
| KL120 | LT174589 | 2.77 | 100 | 16.66 | 100 | 100 | 100 | 100 | 100 | NA |
| KL121 | LT174590 | 16.66 | 100 | 100 | 100 | 100 | NA | 100 | 100 | 100 |
| KL122 | LT174591 | 50 | 100 | 50 | 100 | 100 | NA | 100 | 100 | 100 |
| KL123 | LT174592 | 100 | 100 | 100 | 100 | 100 | NA | 100 | 100 | 100 |
| KL124 | LT174593 | 4.76 | 100 | 100 | 100 | 100 | 100 | 100 | 100 | NA |
| KL125 | LT174594 | 4.16 | 100 | 100 | 100 | 100 | 100 | 100 | 100 | NA |
| KL126 | LT603702 | 50 | 100 | 50 | 100 | 100 | 100 | 100 | NA | NA |
| KL127 | LT603704 | 33.33 | 50 | 12.5 | 100 | 100 | 100 | NA | 100 | NA |
| KL130 | LT603706 | 3.84 | 100 | 100 | 100 | 100 | 100 | 100 | 100 | NA |
| KL131 | LT603707 | 6.66 | 100 | 14.28 | 100 | 100 | 100 | 100 | 100 | NA |
| KL132 | LT603708 | 100 | 100 | 100 | 100 | 100 | NA | 100 | 100 | 100 |
| KL133 | LT603709 | 100 | 100 | 100 | 100 | 100 | NA | 100 | 100 | 100 |
| KL134 | LT603710 | 100 | 100 | 33.33 | 100 | 100 | ``````` | 100 | 100 | 100 |
| KL135 | LT603711 | 5.88 | 100 | 100 | 50 | 100 | 50 | 100 | 100 | NA |
| KL136 | LT603712 | 25 | 100 | 100 | 100 | 100 | NA | 100 | 100 | 100 |
| KL137 | LT603713 | 5 | 100 | 100 | 100 | 100 | 100 | 100 | 100 | NA |
| KL138 | LT603714 | 100 | 100 | 100 | 100 | 100 | 100 | 100 | 100 | NA |
| KL139 | LT603715 | 25 | 100 | 50 | 100 | 100 | NA | 100 | 100 | 100 |
| KL140 | LT603716 | 50 | 100 | 100 | 100 | 100 | NA | 100 | 100 | 100 |
| KL141 | LT603717 | 7.69 | 100 | 100 | 100 | 100 | 100 | 100 | 100 | NA |
| KL142 | LT603718 | 4.76 | 33.33 | 16.66 | 100 | 100 | NA | 100 | 100 | 100 |
| KL143 | LT603719 | 5 | 50 | 100 | 100 | 100 | 100 | 100 | 100 | NA |
| KL144 | LT603720 | 25 | 100 | 100 | 100 | 100 | NA | 100 | 100 | 100 |
| KL146 | LT603721 | 3.84 | 100 | 50 | 50 | 50 | 50 | 100 | 50 | NA |
| KL148 | LT603722 | 3.33 | 100 | 100 | 100 | 100 | NA | 100 | NA | 100 |
| KL149 | LT603723 | 6.66 | 50 | 100 | 100 | 100 | NA | 100 | NA | 100 |
| KL150 | KR007675 | 50 | 50 | 100 | 100 | 100 | 100 | 100 | NA | NA |
| KL153 | LT603725 | 100 | 100 | 100 | 100 | 100 | 100 | 100 | NA | NA |
| KL159 | LT603726 | 4.16 | 50 | 100 | 100 | 100 | NA | 100 | 100 | 100 |
| KN1 | AB924614 | 20 | 50 | 100 | 100 | 100 | 100 | 100 | 100 | NA |
| KN2 | LT174595 | 4 | 50 | 50 | 100 | 100 | 100 | 100 | 100 | NA |
| KN3 | LC189075 | 20 | 100 | 100 | 100 | 100 | NA | 100 | 100 | 100 |
| KL104 | Kaptiveref_KL104 | 4 | 50 | 50 | 50 | 50 | NA | 100 | 100 | 50 |
| KL107 | Kaptiveref_KL107 | 5.88 | 33.33 | 16.66 | 100 | 100 | 100 | NA | NA | NA |
| KL128 | Kaptiveref_KL128 | 3.57 | 50 | 100 | 100 | 100 | 100 | 100 | 100 | NA |
| KL145 | Kaptiveref_Kl145 | 100 | 100 | 100 | 100 | 100 | 100 | 100 | 100 | NA |
| KL147 | Kaptiveref_KL147 | 4 | 50 | 100 | 100 | 100 | NA | 100 | NA | 100 |
| KL151 | Kaptiveref_KL151 | 33.33 | 100 | 100 | 100 | 100 | NA | 100 | 100 | 100 |
| KL152 | Kaptiveref_KL152 | 25 | 100 | 100 | 100 | 100 | NA | 100 | NA | 100 |
| KL154 | Kaptiveref_KL154 | 4.16 | 100 | 50 | 50 | 50 | 50 | 50 | 50 | NA |
| KL155 | Kaptiveref_KL155 | 25 | 50 | 50 | 100 | 100 | NA | 100 | 100 | 100 |
| KL157 | Kaptiveref_KL157 | 100 | 100 | 100 | 100 | 100 | NA | 100 | NA | 100 |
| KL158 | Kaptiveref_KL158 | 5 | 100 | 100 | 100 | 100 | NA | 100 | NA | 100 |
| KL160 | Kaptiveref_KL160 | 50 | 50 | 100 | 100 | 100 | NA | 100 | 100 | 100 |
| KL161 | Kaptiveref_KL161 | 100 | 100 | 100 | 100 | 100 | NA | 100 | NA | 100 |
| KL162 | Kaptiveref_KL162 | 3.03 | 100 | 100 | 100 | 100 | NA | 100 | 100 | 100 |
| KL163 | Kaptiveref_KL163 | 6.25 | 50 | 50 | 50 | 50 | 100 | 100 | 50 | NA |
| KL164 | Kaptiveref_KL164 | 100 | 100 | 100 | 100 | 100 | 100 | 100 | 100 | NA |
| KL165 | Kaptiveref_KL165 | 25 | 100 | 50 | 100 | 100 | 100 | 100 | 100 | NA |
| AVERAGE RELIABILITY SCORE | | 37.13 | 80.83 | 79.88 | 94.28 | 96.43 | 97.18 | 98.54 | 97.52 | 93.81 |

**(B)**


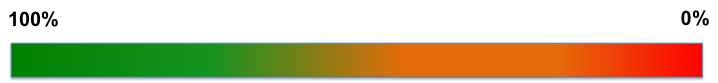


| **Serotype** | **Genbank accession ID (Wzm)** | **Genbank accession ID (Wzt)** | **Reliability Score (%)** | |
| --- | --- | --- | --- | --- |
|  |  |  | **Wzm** | **Wzt** |
| O1 | AAC98411.1 | AAC98413.1 | 33.3 | 33.3 |
| O2 | BAU36938.1 | BAU36939.1 | 33.3 | 33.3 |
| O2aeh | AVA30565.1 | AVA30566.1 | 100 | 100 |
| O2ac | BAU36940.1 | BAU36941.1 | 33.3 | 33.3 |
| O3 | AQZ41253.1 | AQZ41254.1 | 100 | 100 |
| O3 | BAU51051.1 | BAU51052.1 | 50 | 50 |
| O4 | ALX35080.1 | ALX35079.1 | 100 | 100 |
| O5 | BAN20050.1 | BAN20051.1 | 100 | 100 |
| O8 | AAC98405.1 | AAC98406.1 | 100 | 100 |
| O9 | BAU24812.1 | BAU24813.1 | 100 | 100 |
| O12 | BAN08506.1 | BAN08507.1 | 100 | 100 |
| OL101 | CZQ25251.1 | CZQ25252.1 | 100 | 100 |
| OL102 | CZQ25262.1 | CZQ25263.1 | 100 | 100 |
| OL103 | CZQ25266.1 | CZQ25267.1 | 100 | 100 |
| OL104 | CZQ25273.1 | CZQ25274.1 | 50 | 50 |
| Average reliability score |  |  | 83.3 | 83.3 |
