# Supplementary material for: K-PAM: a unified platform to distinguish Klebsiella species K- and O-antigen types, model antigen structures and identify hypervirulent strains
Source: Sci Rep. 2020 Oct 7;10:16732. doi: 10.1038/s41598-020-73360-1 (PMC7541508; doi:10.1038/s41598-020-73360-1)
Supplement: Supplementary file 7 — Supplementary Information 7. [file 41598_2020_73360_MOESM7_ESM.docx]

**K-PAM: A unified platform to distinguish *Klebsiella* species K- and O-antigen types, model antigen structures and identify hypervirulent strains**

L Ponoop Prasad Patro^†^, Karpagam Uma Sudhakar^†^ and Thenmalarchelvi Rathinavelan*

**Table S6.** Test cases corresponding to clinically important *Klebsiella* species whose serotypes are undefined.

| **Wzm Query ID** | **Wzt Query ID** | **Predicted O-type** | | |
| --- | --- | --- | --- | --- |
|  |  | **Wzm** | **Wzt** | **Wzm & Wzt** |
| AAF04380.1 | AAF04381.1 | O5 | O5 | O5 |
| KHF68821.1 | KHF68822.1 | O1, O2, O2ac | O1, O2, O2ac | O1, O2, O2ac |
| EXF40838.1 | EXF40837.1 | O3 | O3 | O3 |
| AAN06492.1 | AAN06493.1 | O12 | O12 | O12 |
| CCI88064.1 | CCI88065.1 | O1, O2, O2ac | O1, O2, O2ac | O1, O2, O2ac |
| CTQ06126.1 | CTQ06130.1 | O3 | O3 | O3 |
| PUH05602.1 | PUH05603.1 | O5 | O5 | O5 |
